# From function to structure: how myofibrillogenesis influences the transverse–axial tubular system development and its peculiarities

**DOI:** 10.3389/fphys.2025.1576133

**Published:** 2025-04-25

**Authors:** Zuzana Sevcikova Tomaskova, Katarina Mackova

**Affiliations:** Department of Biophysics and Electrophysiology, Institute of Molecular Physiology and Genetics, Centre of Biosciences, Slovak Academy of Sciences, Bratislava, Slovakia

**Keywords:** t-tubules, transverse–axial tubular system, postnatal development, cardiomyocyte, sarcomere, Z-line, myofibrillogenesis, costameres

## Abstract

The transverse–axial tubular system (TATS) is the extension of sarcolemma growing to the cell interior, providing sufficient calcium signaling to induce calcium release from sarcoplasmic reticulum cisternae and stimulate the contraction of neighboring myofibrils. Interestingly, the development of TATS is delayed and matures during the *post-partum* period. It starts with small invaginations near the sarcolemma, proceeding to grow an irregular network that is later assembled into the notably transversally oriented tubular network. Accumulating evidence supports the idea that the development of TATS is linked to cell dimensions, calcium signaling, and increasing myofibrillar content orchestrated by electromechanical stimulation. However, the overall mechanism has not yet been described. The topic of this review is the development of TATS with an emphasis on the irregular phase of tubule growth. The traditional models of BIN1-related tubulation are also discussed. We summarized the recently described protein interactions during TATS development, mainly mediated by costameric and sarcomeric proteins, supporting the idea of the coupling sites between TATS and the myofibrils. We hypothesize that the formation and final organization of the tubular system is driven by the simultaneous development of the contractile apparatus under cycling electromechanical stimulus.

## 1 Introduction

An executive agent of cardiac contraction throughout life is muscle cells called *cardiomyocytes*. The synchronous action of cardiomyocytes is described in a process of excitation–contraction coupling (E-C coupling) (Bers, 2002). E-C coupling is allowed by the constitution of Ca^2+^ release units (CRUs), which are junctional domains of the junctional sarcoplasmic reticulum (SR) harboring calcium release channels (ryanodine receptors, RyRs), with sarcolemma harboring L-type Ca^2+^ channels (also known as dihydropyridine receptors, DHPRs) (Franzini-Armstrong et al., 1999). Interestingly, cardiomyocytes are structurally underdeveloped in the *post-partum* period in almost all mammals (Scuderi and Butcher, 2017). A relatively small amount of time is necessary to catch up and build all structural features for mature Ca^2+^ signaling. Sparse myofibrils in a close peripheral position represent the structural conditions for sufficient Ca^2+^ signaling through the sarcolemma (Brook et al., 1983; Hirakow, 1970; Blatter et al., 2003). Different mechanisms of Ca^2+^ signaling have been documented in embryonal, fetal, and postnatal periods in cardiomyocytes (Louch et al., 2015). The cardiomyocyte growth in the postnatal period is marked by the process of switching between hyperplasia and hypertrophy (Li et al., 1996) and other transitional mechanisms (Ostadal et al., 1999; Ikenishi et al., 2012). The initiation of tubular system development could be considered the terminal phase of cardiomyocyte differentiation during growth.

The tubular system was previously referred to as transversal tubules (t-tubules) (Brette and Orchard, 2003) because of prominent transversal orientation in skeletal muscle (Edwards and Launikonis, 2008; Eisenberg and Eisenberg, 1968); nowadays, the preferential name is the transverse–axial tubular system (TATS) (Ferrantini et al., 2013). The TATS is formed to spread the sarcolemma to close apposition with the SR and myofibrils. The main function of the TATS is the conduction of action potential in order to ensure synchronous and efficient Ca^2+^ release from intracellular storage, which triggers cardiomyocyte contraction (Bers, 2002). The functions of the TATS are discussed elsewhere; see Ferrantini et al. (2013), Hong and Shaw (2017), Brette and Orchard (2003), and Lu and Pu (2020). Briefly, Ca^2+^ influx through DHPR channels triggers Ca^2+^ release from RyR2s in the SR, an amplification process known as Ca^2+^-induced Ca^2+^ release (CICR) (Fabiato, 1983). An efficient CICR is enabled by the TATS presence and establishment of special CRUs (dyads), which allows synchronous centripetal propagation of Ca^2+^ transients in ventricular cardiomyocytes (Louch et al., 2010). Delayed Ca^2+^ transients in the cell center and dyssynchronous CICR were observed in cardiomyocytes after formamide detubulation (Brette et al., 2002), whereas, in atrial cardiomyocytes, CICR showed great variability in synchrony, dependent on different distributions of TATS and DHPRs among cardiomyocytes (Frisk et al., 2014). Effective coupling of the CICR mechanism also needs a specific positioning of DHPRs, RyR2s, and other proteins within the dyadic neighborhood (Louch et al., 2010), which needs to be carefully established during development.

The development of the tubular system exhibits some peculiarities. In the rats, the first tubules are observed after the first postnatal week (age postnatal day 7, P7) as small sarcolemmal invaginations (Ziman et al., 2010; Hamaguchi et al., 2013). A sparse and unorganized tubular system is then developed rapidly with a prevalent longitudinal (axial) orientation (Mackova et al., 2017); its prominent transversal orientation is observed after P21 (Han et al., 2013). The irregular growth phase of the tubular network is an unexplained period in the development of cardiomyocytes.

**FIGURE 1:**
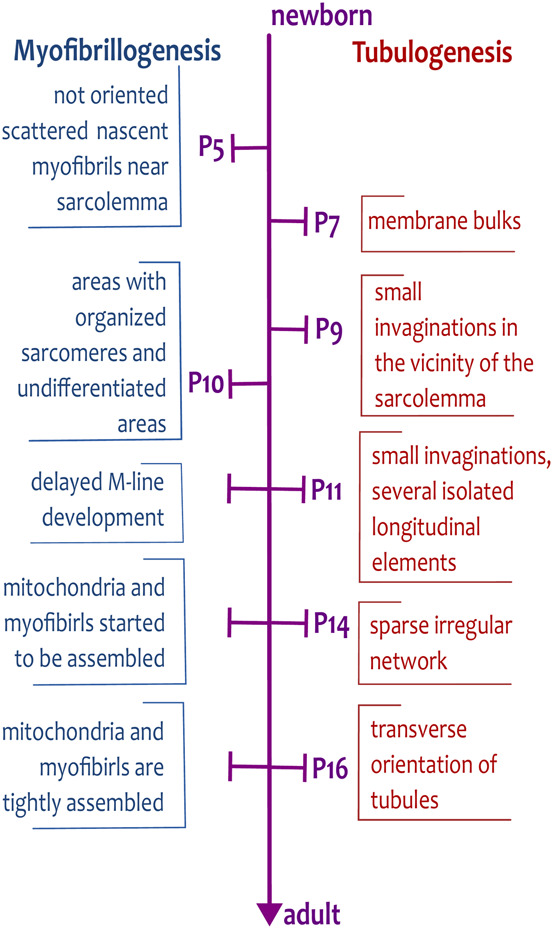
Timescale of myofibrillogenesis and tubulogenesis from newborn to adult stages of rat cardiomyocytes. The important steps of the formation of tubules and myofibrils are indicated on the time scale spanning from the day of birth (P0) to the adult stage (>P21). The development of tubules is delayed and starts on day P7, whereas the first, not oriented, myofibrils are already present during embryonic development.

We review the development of the tubular system, which is characterized by a low degree of organization in the first weeks *post-partum*. During development, the irregularity in the tubular system could relate to the simultaneous process of contractile apparatus development (myofibrillogenesis, sarcomerogenesis; Figure 1). It could be coupled with the cytoskeleton and sarcoplasmic reticulum. Here, we present a hypothesis of tubular system formation driven by contractile apparatus.

## 2 (Im)maturity of induced pluripotent stem cells and engineered heart tissue: key to the coupled developmental mechanisms

In recent years, many laboratories have tried to overcome the immaturity of induced pluripotent stem cell (iPSC) -derived cardiomyocytes (iPSC-CMs) by several mechanisms. One of the highest morphological hallmarks of iPSC-CM maturity is the development of TATS (Giacomelli et al., 2020; Miki et al., 2021; Li et al., 2024) as part of their functional electrophysiological maturity.

The increase in adult troponin I expression via estrogen-related receptor gamma agonist enhances cardiac maturation of iPSC-CMs, including TATS development and enhanced metabolic function (Miki et al., 2021). Combining the Matrigel mattress technique with thyroid hormone triiodothyronine and dexamethasone resulted in human iPSC-CMs exhibiting abundant TATS with synchronized Ca^2+^ release in whole cell volume. Compared with adult cardiomyocytes, TATS was less organized and had more longitudinal elements (Parikh et al., 2017) resembling TATS from the translational postnatal stage (P10–15) of rats (Mackova et al., 2017). It was determined that TATS was developed in line with highly organized sarcomeres with present Z-lines and regularly distributed myofilaments in a long-term 3D culture of iPSC-CMs (more than 50 days) (Ergir et al., 2022). iPSC-CMs seeded in 3D microtissues with cardiac fibroblasts also showed improved sarcomeric structures with transversal tubules, enhanced mitochondrial respiration, and contractility in comparison to iPSC-CMs seeded without fibroblasts (Giacomelli et al., 2020).

The iPSC-CMs seeded onto geometrically engineered substrates exhibited more mature phenotypes like adult CMs, such as elongated cell shape, alignment of myofibrils, and formation of transverse tubules resulting in better electrophysiological properties (Silbernagel et al., 2020; Hong et al., 2023; Khan et al., 2015; Ribeiro et al., 2015). Rectangular 3D scaffolds enabled parallel alignment of myofibrils and the development of tubules in cultured iPSCs. Rectangular-shaped iPSC-CMs showed dense tubular axially oriented invaginations mainly from the shorter poles of the cell. In some cells, the rectangular 3D scaffold induced the development of transverse tubules with a dyadic structure near Z-lines and strong reorganization of DHPR and RyR2 channels relative to each other. Interestingly, the reshaping of the cell from a planar to a rectangular shape also induced a significant height increase that may enable more complex organization of the organelles (Silbernagel et al., 2020). Using a substrate with physiological stiffness (10 kPa) and a rectangular micropattern also promoted increased maturity of iPSC and development of a tubular system (Ribeiro et al., 2015). The distinct mechanical scaffold uses the pillar inside the circular dish, so the iPSC-CMs created the cellular sheet stimulated by a traveling wave originating in a close-loop tissue. Stimulation via looped mechanisms enhanced iPSC-CM maturation, highlighted by the development of a tubular system. However, the tubules are not precisely aligned with the Z-lines (Li et al., 2024).

Engineered heart tissues (EHT) have received much attention recently [see more in Hong et al. (2023)]; EHT can provide higher structural and functional maturation of human iPSC-CMs by several mechanisms. The proper combination of cells with electromechanical stimulation can improve the overall maturation of iPSC-CMs. When early-stage iPSC-CMs (after the onset of assembly of autonomous contraction) were used to engineer heart tissues and were subjected to intense electrical stimulation with periodically increasing frequency, the resulting tissue displayed increased cell size, proper assembly of sarcomeres, and intercalated discs with robust TATS having prominent transverse components and functional CRUs. Interestingly, this robust maturation of iPSC-CMs was achieved only by physical conditioning with increasing intensity of mechanical stimulation that mimicked mechanical loading during the perinatal heart development (Ronaldson-Bouchard et al., 2018). The tubular system, with a pronounced axial component, was present in electro-mechanically stimulated EHTs created from neonatal rat cardiac cells, comparable to TATS observed from P13 isolated cardiomyocytes (Godier-Furnémont et al., 2015).

Kermani et al. (2023) recently reported that seeding of iPSC-CMs in cuboid 3D micro-scaffolds and overexpression of amphiphysin2 (BIN1) led to increased tubulogenesis and improved Ca^2+^ handling at the level of E-C coupling. This finding opens new avenues for future human iPSC-CM development and applications based on a combination of engineering approaches with knowledge of complex coupled developmental processes.

These techniques have unmasked tremendous coupled mechanisms involved in cardiac maturation. It certainly seems that mechanical stimulation, such as contraction, must be present to promote the growth of TATS.

## 3 Cardiomyocyte growth in the postnatal period

Heart development is also fueled by mechanical forces. The heart is the first organ developed during embryonic development and starts to beat in an autonomous manner between embryonic days 8–9 (E8–E9) in mice (Chen et al., 2010). At birth, the mammalian heart is not fully mature. The overall cardiac parameters rapidly increase in the *post-partum* period (Papanicolaou et al., 2012). The increased workload is manifested by an increase in both pressure and volume load, which causes the enlargement of ventricular volume and the increase of wall thickness (Olivetti et al., 1980).

**FIGURE 2:**
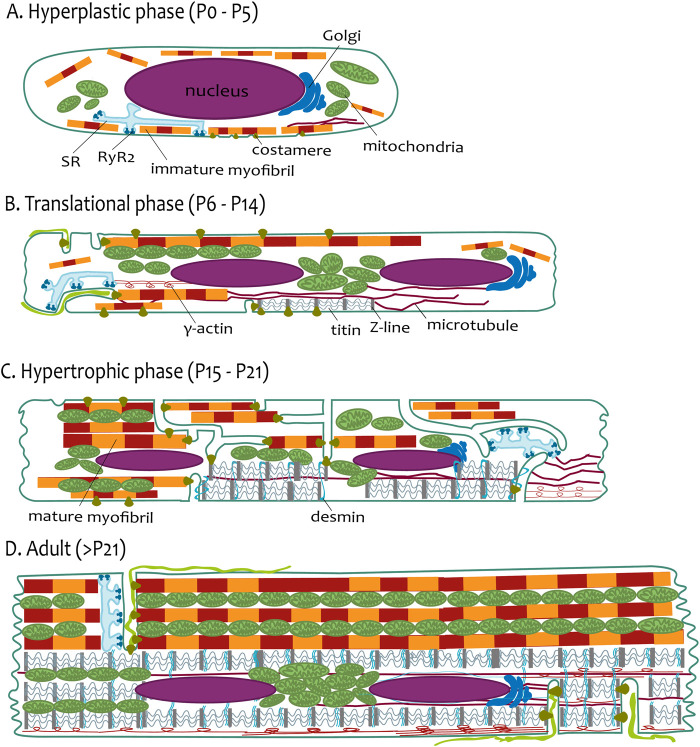
The phases of postnatal growth of the rat ventricular cardiomyocyte. **(A)** Hyperplastic phase (P0–P5). The cardiomyocyte has a spindle-like shape with a prominent nucleus. The immature myofibrils are localized near sarcolemma or loosely distributed in the cytoplasm. SR creates the CRUs (periphery couplings) with the sarcolemma. **(B)** Translational phase (P6–P14). The cardiomyocyte has a more rectangular-like shape. Two nuclei are already present in most of the cells. Short tubules are present coupled with myofibrils via costameres and longer tubules coupled with more mature myofibrils. The SR creates the CRUs with this longitudinal tubule. The scaffold of cardiomyocytes is emphasized with a Z-line connected with titin filaments surrounded by a developing network of microtubules and γ-actin. **(C)** Hypertrophic phase (P15–P21). The cardiomyocytes resembles the adults. The myofibrils are almost the cell volume, although they are not fully laterally aligned. TATS creates a sparse and irregular network. CRUs are present at the transversal and longitudinal elements of TATS. The transversal alignment of the tubules has started and is mediated by desmin. **(D)** Adult (>P21). The cardiomyocyte has a rectangular shape; the proportions of the cellular components are schematic. The aligned mature myofibrils are interwoven with the rows of mitochondria. The TATS has a prominent transversal orientation with well-developed CRUs (dyads). The scaffold of cardiomyocytes is finished by the maturation of sarcomeres and lateral alignment of all myofibrils mediated by desmin.

The cardiomyocyte growth is executed in three phases of growth in newborn rat hearts (Figures 1, 2): i) an early phase of hyperplasia (i.e., cell division) in postnatal days P0 to P5; ii) a transitional phase from day P6 to P14; and iii) a hypertrophic phase between days P15 and P21 (Clubb and Bishop, 1984). After P21, the rat reaches the adult phase.

On the ultrastructural level, cardiomyocytes are not fully assembled during the postnatal period (Hirakow, 1970; Brook et al., 1983; Anversa et al., 1980). The early postnatal period (P0–P5; Figure 2A) is mainly focused on the beginning of the binucleation process and protein expression mirrored on organelle structure. In rats, during P1–P3, cardiomyocytes are mononucleated with constant cell volume. The binucleation starts after P4, and most cardiomyocytes (90%) become binucleated at P12 (Li et al., 1996). The shape of mouse cardiomyocytes changes from round to polygonal during embryonic stages and *post-partum* to spindle-like shapes at P4. Prominent empty cytoplasm with scattered mitochondria around the nucleus is seen in P3 mouse cardiomyocytes (Piquereau et al., 2010). Myofibrillogenesis begins in the early embryonic period and continues in the postnatal period. The nascent myofibrils are not oriented and occupy little space between the sarcolemma and the massive nuclei in embryonic mice cardiomyocytes (Hirschy et al., 2006; Legato, 1979; Brook et al., 1983). Prominent cell growth in the longitudinal direction parallels myofibril elongation, and costameric regions are formed on the lateral membrane (Hirschy et al., 2006).

The myofibrillogenesis continues during the translational growth phase (P6–P14; Figure 2B). In mouse cardiomyocytes, myofibrillar bundles acquire the proper longitudinal orientation at P7 (Papanicolaou et al., 2012). The rat ventricular myocardium during P10 has areas with properly organized sarcomeres but also undifferentiated areas (Hopkins et al., 1973). Delayed development of the M-line of myofibrils was observed in rat myocardium, which is clearly demonstrated after P11 (Anversa et al., 1981). Costameric regions, manifested by laminin, are localized on lateral membranes in P10 mouse cardiomyocytes (Hirschy et al., 2006). At P14, mouse cardiomyocytes are cylindrical, with well-developed cell-to-cell connections and mature intercalary disks (Wilson et al., 2014). The tubular system is present as irregular invaginations at this period.

The hypertrophic phase (>P14; Figure 2C) is marked by proper structural assembly, resembling the adult condition (Hopkins et al., 1973). Mitochondria with myofibrils are tightly assembled toward P21 (Piquereau et al., 2010). Interestingly, a short but intense proliferative burst of cardiomyocytes at P15 in the mouse left ventricle (Naqvi et al., 2014) could point out more flexible postnatal developmental processes, including myofibrillogenesis, following heart output demands (Figure 2C).

Not only ultrastructural changes but also protein expression changes and metabolic changes occur during this period (Puente et al., 2014; Bartelds et al., 2000; Hoerter et al., 1991; Vornanen, 1996), ultimately leading to the efficient E-C coupling with fully assembled CRUs and optimally organized TATS (Flucher and Franzini-Armstrong, 1996) in mature cardiomyocytes (Figure 2D).

## 4 Development of the transverse–axial tubular system

The TATS is developed later than other structures engaged in E-C coupling; myofibrils and SR are detected during embryonic development. TATS is observed for the first time after P7 in ventricular cardiomyocytes of mice (Hamaguchi et al., 2013; Chen et al., 2013) and rats (Ziman et al., 2010; Mackova et al., 2017), with no dependency on origin from right or left ventricles (Chen et al., 2013).

In rodent ventricular cardiomyocytes, the development of a tubular system occurs between P7 and P20 (Figure 2). At first, the early tubules appear on P7 as small membrane bulks. On P9, tubules are formed by small invaginations in the vicinity of the sarcolemma, with approximately 2.0 µm length. On P11, tubules form not only short invaginations but also several isolated longitudinal elements (Figure 2B). Later, on P14–P16, tubules create a sparse irregular network (Figure 2C) that reaches a higher degree of organization after day P16 (Mackova et al., 2017; Ziman et al., 2010). The regular organization of the tubular system, with prominent transversal orientation and proper intertubular distance, appears after P20 (Han et al., 2013). Similarly to cardiomyocyte growth phases, one can define phases of TATS development: i) early phase of invagination (P7–P9); ii) transitional phase of irregular growth from first longitudinal elements to the branched tubular system (P10–P15); iii) phase of arrangement to the dominant transversal orientation (≥P16); Figures 1, 2.

**FIGURE 3:**
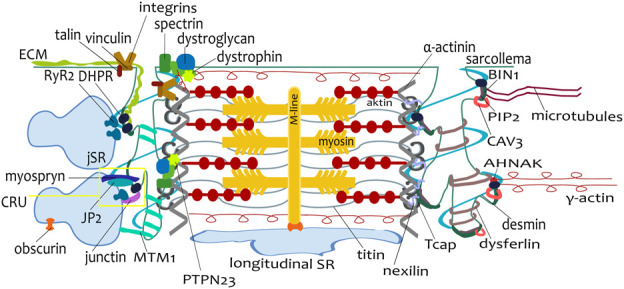
Scheme of a single sarcomere with proteins involved in tubulogenesis and myofibrillogenesis. The connection between tubules and sarcoplasmic reticulum, including the calcium release unit (CRU, dyad), is visualized on the left side of the sarcomere. The connection of a tubule to a Z-line, mediated by many different proteins, is depicted on both tubules. During postnatal development, the expression of these proteins varies according to the developmental stage.

An important part of tubular system development is the assembly of CRUs (Figure 3). At first, the periphery couplings are formed by the close position of junctional SR and the sarcolemma (Sun et al., 1995); their number increases, and their formation is followed by the development of dyads (Franzini-Armstrong et al., 2005). In the guinea pig heart, the periphery couplings shift continually deeper into the cell core with tubule growth (Forbes and Sperelakis, 1976). In general, the docking of junction membranes was specified as the first step of CRU formation. At the time of docking, the sarcolemma does not contain an efficient amount of DHPR clusters, and the SR contains few RyR channels; these are delivered later. The formation of CRU is completed with the assembly of DHPR and RyR channels to target membranes as well as with the assembly of complex RyR with calsequestrin, with supporting molecules triadin and junctin in the SR (Franzini-Armstrong et al., 2005). Interestingly, the membranes of the sarcolemma and SR have the capacity to form junctions in the absence of DHPRs (Powell et al., 1996), and RyRs are not essential for membrane junctions (Takeshima et al., 1995; Takekura et al., 1995). Taken together, CRU formation is a very important part of tubule development and functionality, although it is not essential for tubule biogenesis and growth.

### 4.1 Tubulation of sarcolemma

Three possible mechanisms for tubule formation have been proposed: formation of tubules by sarcolemmal invagination, additions of membrane vesicles via continuous fusion, or initial formation of the internal network, which later will be connected to the sarcolemma (Flucher et al., 1993). Ferritin-based tracing of the extracellular space revealed 72%–88% of ferritin-filled tubule/caveolae profiles in P2–P3, which were well-observed in P14 rat cardiomyocytes (Di Maio et al., 2007). The remaining observed profiles were called immature dyads, formed by tandem vesicles containing junctional RyRs and DHPRs but not connected to the sarcolemma. Most tubules grow inward to the cell, connected to the sarcolemma (Forbes and Sperelakis, 1976; Hirakow, 1970; Franzini-Armstrong, 1991; Hagopian and Nunez, 1972), with the minor addition of prearranged tandem vesicles of DHPRs and RyRs (Di Maio et al., 2007).

The initial step is not yet understood despite several proposed mechanisms of membrane curvature induction reviewed by Zimmerberg and Kozlov (2006). The protein superfamily containing the BAR domain has recently received much attention. The BAR domain is a crescent-shaped dimer that binds to the negatively charged membrane (Peter et al., 2004). The scaffolding protein amphiphysin2 (BIN1; Figure 3) contains the BAR domain and can induce membrane curvature (Becalska et al., 2013). In Chinese hamster ovary cells and myoblastic cell line C2H12, the extensive tubular system was revealed with fluorescently tagged BIN1 (Lee et al., 2002). Expression of BIN1 induced short invaginations in C2C12 myoblasts, whereas co-expression of myotubularin 1 (MTM1; Figure 3) with BIN1 led to longer tubules creating a network (Royer et al., 2013). The BIN1 is important in the process of tubulation, as is seen from the studies of BIN1-knockout *Drosophila* skeletal myocytes (Razzaq et al., 2001) and mouse cardiomyocytes (Hong et al., 2014), which showed disruption of the tubular system. Moreover, it was proposed that BIN1 localizes DHPR in the tubule membrane structure and enables its targeted delivery.

Targeted delivery of DHPR-containing vesicles throughout a dynamic microtubule network, tethered with a BIN1 membrane scaffold, was observed in non-myocyte HeLa and HL-1 cell lines (Hong et al., 2010). A potential association of actin filaments with the trafficking of DHPRs from the perinuclear area to the tubular system was indicated in a study on cultivated adult atrial cardiomyocytes (Leach et al., 2005). In human and mouse left-ventricular cardiomyocytes, BIN1 colocalized with DHPR in the typical transversal organization and created protective inner folds of the membrane. The loss of the BIN1 scaffold in BIN1 knockout mice was manifested by prolongation of the action potential and increased probability of ventricular arrhythmia (Hong et al., 2014). Overexpression of BIN1 in human embryonic stem cell-derived cardiomyocytes showed induction of TATS growth with DHPR clustering along the tubules. With increased expression of BIN1, the length and width of tubules also grew (De La Mata et al., 2019).

Lipid phosphatidylinositol-4,5-bisphosphate (PIP2) was revealed as being responsible for the localization of BIN1 to the sarcolemma. Damaged BIN1 organization was induced in cardiomyocytes with heart failure without reduced protein levels when PIP2 was not present to provide BIN1 membrane insertion (Zhou et al., 2021). Interestingly, a recent study of murine ventricular cardiomyocytes in P10 showed preceding localization of BIN1 in a regular, transverse pattern along Z-lines consistent with future positions where TATS will grow (Perdreau-Dahl et al., 2023). The study also observed that in both mouse cardiomyocytes and human iPSC-CMs, BIN1-dependent tubulogenesis is regulated in opposing directions by MTM1 and dynamin 2. A high level of MTM1 is essential for enabling BIN1 to induce the growth of TATS. Dynamin 2, in contrast, plays an inhibitory role, explaining declining expression levels during postnatal maturation. Thus, these three proteins jointly regulate cardiomyocyte t-tubule growth (Perdreau-Dahl et al., 2023).

The hypothesis of budding caveolae (Razani et al., 2002) was considered to be the possible developmental mechanism observed in chick skeletal muscle *in vitro* (Ishikawa, 1968). The observation agrees with the increasing amount of the major protein of caveolae—caveolin 3 (CAV3; Figure 3) in the tubular membrane during development (Parton et al., 1997). This observation can be partially challenged by the function of caveolae to respond to acute mechanical stress (Sinha et al., 2011). The caveolae have a mechanoprotective role from rupture during cardiac output (Cheng et al., 2015). The increasing CAV3 abundance could be associated with increased mechanical stress and load during development. Their engagement in tubule growth is a matter of question, as the TATS was observed, despite its disorganization, in CAV3 knockout mice skeletal myocytes (Galbiati et al., 2001) and in human skeletal muscle cells with the *CAV3* gene mutation (Minetti et al., 2002).

Recently, novel structures that were termed caveolae rings, formed by CAV3 and BIN1, were discovered in differentiated primary murine and human skeletal myotubes. Caveolae rings are formed on BIN1 scaffolds that create contact sites with RyR1-positive SR cisternae, are enriched in DHPR, and act as a platform for tubule formation. In addition, the BIN1-induced membrane tubulation requires proper CAV3 function. This would suggest that caveolae rings are the sites for tubule initiation and elongation (Lemerle et al., 2023).

Another protein, dysferlin (DYSF; Figure 3), is suspected to have a role in the elongation of tubules. Dysferlin is a transmembrane protein localized on sarcolemma and the tubular membrane of muscle cells (Klinge et al., 2010). Moreover, the interaction between DPHRs, CAV3, and DYSF was shown by immunoprecipitation (Ampong et al., 2005). The DYSF protein is a member of the ferlin protein family. It possesses several C2-domains for linking to phospholipids, which could facilitate vesicle fusion and membrane repair (Cárdenas et al., 2016). While DYSF can induce membrane tubulation, other ferlin proteins, like myoferlin, have not. This suggests the unique role of DYSF within the ferlin family (Hofhuis et al., 2017). Interestingly, DYSF is associated with the development of the tubular system in neonatal skeletal myocytes (Klinge et al., 2010) and in non-muscle cells (Hofhuis et al., 2017). In a study of regenerated skeletal muscle *in vivo*, DYSF was temporarily localized in the cytoplasm during the regenerating phase. Otherwise, DYSF colocalized with DHPRs in tubules. The tubules were dominantly longitudinally oriented 4 days after muscle injury. By the seventh day, DHPR- and DYSF-labeled tubules were both transversal and longitudinal. DYSF relocated to the sarcolemma 10 days after injury (Klinge et al., 2010). As was proposed before, DYSF could contribute to resealing the injured sarcolemma (Bansal et al., 2003). DYSF-deficient skeletal muscle had an abnormal tubular system with dilated lumen and irregular or longitudinal orientation (Klinge et al., 2010). Moreover, DYSF is associated with the prevention and stabilization of stress-induced Ca^2+^ signaling in the tubular system (Kerr et al., 2013) and decreases the damage of the tubular membrane by glycerol in the skeletal muscle (Demonbreun et al., 2014). These observations suggest an additional supportive role of DYSF protein.

The shifting of the newly formed tubular system to an organized transversal orientation might also be mediated by other protein interactions. One such protein is juncthophilin2 (JP2; Figure 3), a protein that spans the membranes of the sarcolemma and sarcoplasmic reticulum (Takeshima et al., 2000). On day P10, JP2 colocalizes with the sarcolemma, and this distribution changes during tubular system development. The diminishing distribution of JP2 on the periphery and its simultaneously increasing localization on tubules could be connected with the process of dyad assembly (Ziman et al., 2010). The expression of JP2 is detectable during P4, with a nearly 4-fold increase of expression at P8. In murine myocardium, the expression of JP2 reached the adult level at P14 (Chen et al., 2013).

Late disorganization of the TATS, with dominant longitudinal orientation, was described in JP2 knockout mice (Han et al., 2013). Moreover, the JP2 deficiency caused a decrease in the density of transversal tubules with no effect on the longitudinal elements. JP2 probably has a secondary role in the tethering of tubules to the cisternae of the sarcoplasmic reticulum, while the longitudinal tubules seem to have JP2-independent regulation (Chen et al., 2013). The role of JP2 in the anchorage of tubules to SR and in the tubular organization was demonstrated by a mouse model with overexpression of JP2 in the heart. Whereas at P5, there was no difference in tubular organization, the tubular growth in myocardium with overexpressed JP2 was accelerated at P8 (Reynolds et al., 2013).

Apart from JP2, other proteins were shown to be engaged in the process of transversal orientation of TATS. Royer et al. (2013) suggested that MTM1 regulates transversal localization of BIN1 during maturation. BIN1 lost clear transversal tubule doublet localization in the skeletal muscle of *Mtm1* knockout mice (Royer et al., 2013). Junctin (Figure 3) is a membrane protein that binds to RyR, calsequestrin, and triadin in junctional SR. Overexpression of junctin in mouse cardiomyocytes leads to an increased association of SR with tubules in terms of both the number of interactions and the size of the interacting area (Zhang et al., 2001). Junctin could have a role in the deposition of tubules in transversal orientation, which is supported by the significant increase in the expression of junctin during the postnatal period in rabbit myocardium (Wetzel et al., 2000). However, in the study of P7 and P14 old mouse myocardium with overexpression of junctin, calsequestrin, or both, detailed analysis of phenotypes revealed only a minor effect of junctin for docking tubular membrane to junctional SR (Tijskens et al., 2003).

## 5 Longitudinal and irregular tubules in adult cardiomyocyte

In rat adult left-ventricular cardiomyocytes, the tubular system comprises approximately 3.6% of cellular volume. The prominent organization of the tubular system has a transversal direction, but tubules occurring also in close proximity to the Z-lines have been observed (Soeller and Cannell, 1999), along with less abundant longitudinal or oblique elements and branches (Ogata and Araki, 1994). Some studies also mentioned oddly shaped tubules during development: ampulla-like dilatation beneath plasmalemma, oblique tubules with a rib-like profile, flattened cisternae (Nakamura et al., 1986), or vesicular-like structures (Dan et al., 2007). Recently, more tubular shapes were described in a study of TATS sheep atrial cardiomyocytes recovered after heart failure induced by fast ventricular pacing. Most transversally oriented tubules were supplemented with longitudinal and more branched tubules in recovered cardiomyocytes. Additionally, the tubules with angled, stumped, cactus-like pairs, oak-tree-like, or lattice shapes were described (Caldwell et al., 2024). The average diameter of a tubule is 200–300 nm, with a high variability from 45 nm to 450 nm (Soeller and Cannell, 1999). The variability in diameter can be explained by the unusual tubule shape or simply by the local tubule position if it is located in a narrow space between myofibrils or between a myofibril and mitochondria (Bennett et al., 2016). The dilatation of tubules near dyadic junctions was described, possibly due to regional specialization for the depletion of tubular lumen (Wong et al., 2013). The tubules consist of extensive microfolds. It has been proposed that these microfolds are formed by the BIN1 protein (Hong and Shaw, 2017).

Whereas transversal tubules are engaged in spreading Ca^2+^ transients through dyads, the role of longitudinal or irregular tubules in mature cardiomyocytes is blurred. Longitudinal tubules run along the myofibrils and at least along one sarcomere (Wagner et al., 2014; Sperelakis and Rubio, 1971). These tubules can also be at a close distance to the nucleus, often bridging several neighboring sarcomeres (Jayasinghe et al., 2010). Not every observed longitudinal element is the result of branching of adjacent transversal tubules; it could also be a single long sinuous tubule penetrating to the cytoplasm (Pinali et al., 2013).

Longitudinal elements of the tubular system can have a different role than the transversal elements. JP2 deficiency caused interrupted maturation of transversal elements but had no effect on the density of longitudinal elements, probably due to their distinct regulation by JP2 (Chen et al., 2013). A study of frog skeletal muscle (Voigt and Dauber, 2004) revealed longitudinal or tangled tubules in myofibril-free cytoplasm near nuclei, Golgi apparatus, and mitochondria. The involvement in the depletion of metabolic products has been suggested as a distinct function of the tubular system, unrelated to E-C coupling. On the other hand, it has been reported that action potential propagates also through longitudinal elements in skeletal muscle. It may be important in the reduction of possible action potential failure (Posterino et al., 2000). The propagation velocity of the action potential is approximately 25% slower in longitudinal tubules than in the transversal (Edwards et al., 2012). Almost 75% of longitudinal tubules created longitudinal junctions with adjacent SR, having an average length of 510 nm and undistinguishable structure from dyads on transversal tubules (Asghari et al., 2009). These observations suggest that dyads on longitudinal tubules are functionally involved in the process of E-C coupling (Das and Hoshijima, 2013). The simulation of the effect of tubular orientation on calcium transients revealed that the low density of transversal tubules, with at least a few longitudinal tubules, ensures centripetal wave propagation (Marchena and Echebarria, 2020).

The function of the tubular system with dominant axial orientation is documented in rat atrial cardiomyocytes. If the tubular system was present, it appeared as reticular, with a low degree of organization and strong longitudinal orientation. Simultaneous labeling of the sarcolemma and Ca^2+^ release showed two types of Ca^2+^ transients. The presence of Ca^2+^ sparks was associated with the localization of these longitudinal tubules, which was in line with the distinct shapes of Ca^2+^ transients (Kirk et al., 2003). Brandenburg et al. (2016) studied TATS in the murine heart using super-resolution confocal microscopy and electron microscopy. The central axial tubular structure, with remarkably large width and surface area, exhibited high levels of DHPR clusters and extensive SR junctions. Surprisingly, the observed onset of a Ca^2+^ transient on central axial tubules was faster than on peripheral sites. The authors also demonstrated that the proliferation of the tubular system is an effect of atrial hypertrophy (Brandenburg et al., 2016). The accelerated Ca^2+^ release from RyR2 clusters was associated with their faster activation due to more abundant longitudinal tubules in thicker atrial cardiomyocytes.

### 5.1 Tubules under pathological conditions

The longitudinal or disorganized elements of the tubular system receive much more attention during pathological conditions. An increased incidence of longitudinal tubules was observed in relation to the loss of cross-striation and misalignment of myofibrils in the denervated rat skeletal muscle (Takekura and Kasuga, 1999) as well as in skeletal myocytes affected by congenital muscular dystrophy (Miike et al., 1984). A remodeled tubular system was observed in post-myocardial infarcted rabbit cardiomyocytes. In the border zone of infarction, tubules were altered and created “t-sheets,” with the structure dilated in the longitudinal direction and at the same time connected to the sarcolemma (Seidel et al., 2017b). A similar structure was described at the end stage of human heart failure (Seidel et al., 2017a). The regional differences after myocardial infarction (MI) in sheep hearts were observed: TATS were fragmented, sparse, and irregular in the border zone of MI, whereas in remote regions, TATS was slightly fragmented, not significantly different from the control (Perera et al., 2022). Disease-related structural abnormalities of TATS (Crocini et al., 2017) also include a reduction in the number of tubular openings to the sarcolemma (Lyon et al., 2009) or increased average diameter and length of tubular elements (Ibrahim et al., 2012a; Wagner et al., 2012).

Importantly, disorganized TATS leads to pathological functionality. Graded changes in mouse ventricles after aortic constriction were connected with graded cellular hypertrophy, TATS loss, decreased expression of JP2 and CAV3, and decreased L-type Ca^2+^ current density (Bryant et al., 2018). Loss of TATS regularity and dyads during heart failure leads to the presence of orphaned RyR2s and insufficient Ca^2+^ transients, which in turn causes slower contraction and reduced cardiac force (Song et al., 2006). The dyssynchronization of local Ca^2+^ release contributes to the broadening and slowdown of the overall Ca^2+^ transient in heart failure (Lyon et al., 2009). It has been suggested that the reliance of Ca^2+^ release on TATS density even accelerates as TATS remodeling increases during conditions of heart failure (Yamakawa et al., 2021). It was also shown that in a rat model of post-ischemic heart failure, some elements of disorganized TATS exhibit abnormal electrical activity, which leads to failure of action potential propagation. These failing tubular elements can fire local spontaneous depolarizations without propagation to the whole TATS (Sacconi et al., 2012), which can sometimes trigger local Ca^2+^ release as a possible new arrhythmogenic phenomenon (Crocini et al., 2016a). Interestingly, the tubules failing to propagate action potential represented a significant proportion of all tubules and caused abnormalities of Ca^2+^ transient even without overall TATS network changes in the model of hypertrophic cardiomyopathy (Crocini et al., 2016b). Recently, the loss of TATS structures during heart failure led to a redistribution of L-type Ca^2+^ channels to the “sarcolemmal crests,” which caused their hyperactivity and more frequent spontaneous Ca^2+^ release (Medvedev et al., 2021; Sanchez-Alonso et al., 2016).

The development of dyadic structures remarkably resembles the reverse process of dyad unpacking during heart failure, as discussed by Lipsett et al. (2019). They stated that the resemblance is just apparent; the developing tubular elements are functional shortly after growing, whereas failing tubules and dyads are functionally deficient in conditions of heart failure (Lipsett et al., 2019). As was mentioned previously, irregularities of TATS were consistently observed during cardiac development, with structural similarities of TATS during various pathological conditions. It is not clear whether the underlying mechanism is the same under developmental and pathophysiological conditions, even though the “last in, first out” paradigm of Lipsett and colleagues sounds appealing.

## 6 Structure follows function: Why does TATS even exist?

The organization of the tubular system is directly connected to the state and function of myocytes (Watson et al., 2016). The rudimentary tubular system was described in rat atrial cardiomyocytes (Bootman et al., 2006); however, several recent studies observed a more abundant tubular system in murine (Brandenburg et al., 2016) and rat (Frisk et al., 2014) atrias. The variability of the atrial tubular system in rats was associated with tissue variability: approximately 30% of atrial cardiomyocytes possessed a tubular system, of which 10% was well-organized (Frisk et al., 2014). In higher mammals, like sheep (Dibb et al., 2009), atrial cardiomyocytes were observed to have a tubular system with a prominent longitudinal orientation. The left ventricular tubular system is tricky as well. Highly reticular patterns and 4-fold higher tubular density were observed in rat and mouse ventricles, whereas a simpler, beam-like tubular system was observed in higher mammals such as humans or horses (Jayasinghe et al., 2015). In amphibians (Galli et al., 2006) or birds (Jewett et al., 1971), the TATS is not formed at all. High variability of the tubular system within myocytes relates to the surface-to-volume ratio, cardiomyocyte dimensions, myofibrillar content, and cardiac frequency (Hirakow, 1970). The structure of the tubular system is highly dynamic and able to react, change, and grow according to the cell's needs (Ferrantini et al., 2013).

With the formation of the tubular system, Ca^2+^ transients have faster kinetics in the central part of the cell, thanks to the development of dyads (Tohse et al., 2004). The requirement for tubule development could be associated with the increasing cell diameter. The tubular system is not present in a cell with a smaller diameter because the amount of Ca^2+^ influx through the sarcolemma is sufficient for contraction (Bers, 2001). According to a model of centripetal Ca^2+^ diffusion in cardiomyocytes, it is apparent that the contribution of tubules to Ca^2+^ synchrony is more critical with increased cell diameter (Gadeberg et al., 2016). The diameter of cardiomyocytes is relatively constant in the first postnatal week, with the increase in diameter observed after P11 (Mackova et al., 2017; Vornanen, 1996). In the mammalian left ventricle, the tubules start to grow when a critical cardiomyocyte diameter of ∼7 µm is reached (Hirakow, 1970). Hence, every fiber of contractile apparatus in myocardial ventricular myocytes has, in close distance of up to ∼1 μm, either a longitudinal or a transversal tubule (Sperelakis and Rubio, 1971). A strong relationship between cell dimensions, Ca^2+^ signaling, myofibrillar content, and development of the tubular system is apparent. More and more studies reveal close mechanic relationships between “excitation apparatus (tubules, SR)” and contractile apparatus (myofibrils) (Stanczyk et al., 2018; Wilson et al., 2014; Bennett et al., 2016; Kostin et al., 1998).

The developmental processes are tightly coupled with increased mechanical requirements. Sheldon et al. (1976) postulated the idea of mutual development of TATS and myofibrils. The electron microscopy scans of lamb left ventricles from different gestation phases (90–110 days) showed clear physical attachment of tubules with Z-lines during myofibrillogenesis. During the splitting and branching of myofibril, the adherence of the tubule allowed it to follow the Z-line, which induced the transversal growth of the attached tubule. The axial growth of attached tubules was induced by the occasional elongation of the myofibril (Sheldon et al., 1976).

## 7 Coupling of TATS and myofibrils

The tubules are attached to the myofibrils, as was observed on electron microscopy images (Brook et al., 1983; Anversa et al., 1981; Hirakow, 1970; Nassar et al., 1987; Sheldon et al., 1976). There are many candidates for the connection of tubules and myofibrils (Figure 3). We summarize the proteins that could be involved in this process because their function, localization, or expression profile correlates with tubule development.

### 7.1 Proteins of extracellular matrix and costameric proteins

The tubular system has a basal lamina formed by proteins of the extracellular matrix (ECM), mainly laminin and collagen. In idiopathic dilated cardiomyopathy, the increased level of collagen type IV in the lumen of tubules caused their increasing diameter (Crossman et al., 2017). The proteins of ECM are also assembled during days P2-P20 in rat and hamster cardiomyocytes (Borg, 1982), which is strongly correlated with TATS development.

The myofibrils are attached to the ECM (through sarcolemma) by structures called costameres (Ervasti, 2003). Two major protein complexes have been identified at costameres: the dystrophin–glycoprotein complex (DGC) and the integrin-talin-vinculin complex; see more in Peter et al. (2011). It was proposed that costameric proteins are important for the mechanical stability of the tubular system (Kostin et al., 1998).

Dystrophin is a subsarcolemmal protein (Figure 3) involved in the mechanical stability of the membrane (Woolf et al., 2006). It is present in the tubular system of human (Kaprielian et al., 2000), sheep, and rabbit cardiomyocytes (Klietsch et al., 1993) and is associated with the regulation of DHPR channels (Sadeghi et al., 2002; Woolf et al., 2006; Koenig et al., 2013). Analysis of dystrophin mRNA transcripts revealed distinct isoforms of protein in different stages of development and in adulthood (Bies et al., 1992). The early expression of dystrophin has an essential role in the assembling of costameres during fetal development of skeletal and cardiac muscle (Chevron et al., 1994). The distribution of dystrophin correlated with the development of the tubular system in rabbit cardiomyocytes: dystrophin was on the lateral sarcolemma at P4, when tubules were absent, whereas, at P7, dystrophin was localized on short tubular invaginations (Frank et al., 1994).

Recently, protein PTPN23 (tyrosine phosphatase, nonreceptor type 23; Figure 3) was identified as a new dystrophin-associated protein that could coordinate the formation of TATS and their attachment to Z-lines (Xu et al., 2024). Immunostaining of CAV3 and JP2 confirmed postnatal patterning: a lack of colocalization with PTPN23 in P5 cardiomyocytes, but PTPN23 partially overlapped with CAV3 and JP2 at P10. The genetic deletion of PTPN23, α-actinin, or dystrophin resulted in similar defects in TATS patterning. In each model, tubules were enlarged, became less organized, and were more longitudinally oriented. The hypothesis was proposed that the recruitment of PTPN23 to Z-lines is required for the assembly of the DGC complex and to anchor tubules to the sarcomeres (Xu et al., 2024).

Dystroglycan is a transmembrane receptor linking the ECM to the cell membrane (Figure 3), which is present together with matriglycan within TATS (Klietsch et al., 1993). Recently, it has been shown that mice with defects in glycosylation of dystroglycan developed normal TATS but were susceptible to stress-induced TATS loss. Similar stress-induced cardiac TATS disruption was observed in a cohort of mice that solely lacked matriglycan. The data indicate that dystroglycan in TATS anchors the luminal ECM to the tubular membrane via the polysaccharide matriglycan, which provides the TATS resistance to mechanical stress and prevents disruptions in TATS integrity (Hord et al., 2024).

Spectrin is the main component of the membrane skeleton (Figure 3), which permits withstanding very strong mechanical stresses (Machnicka et al., 2014). Spectrins are flexible rods with binding sites for F-actin at each end (Bennett and Healy, 2008). Their βII isoform forms complexes with ankyrins and actins (Derbala et al., 2017; Kee et al., 2009). In CRUs, spectrin is one of the spanning molecules between the tubular membrane and SR (Mohler et al., 2005). However, there was no difference in DHPR localization or TATS structure in the βII spectrin conditional KO mice (Smith et al., 2015). Postnatal localization of spectrin in hamster cardiomyocytes changes continually: at P5, immunolabeled spectrin was localized on the sarcolemma and short invaginations, and it progressively moved transversally in the striated pattern (Messina and Lemanski, 1991). In mice with dilated cardiomyopathy, the elongation of myofibrils was observed at the top of the intercalated disc folds. The spectrin-rich loops in this area were enlarged and often associated with junctional SR vesicles, which could be future CRUs on transversal tubules at the Z-line after the insertion of a new sarcomere (Wilson et al., 2014). Spectrin also facilitates the development of branched and dilated tubules after osmotic shock in some cells (Herring et al., 2000), which supports the role of spectrin in the development of the tubular system in cardiomyocytes (Messina and Lemanski, 1991).

Vinculin is a 117-kDa membrane-associated protein that is present as a key component of costameres and intercalated disks (Tangney et al., 2013). Vinculin has a role in the attachment of myofibrils to the sarcolemma and TATS (Pardo et al., 1983). It is present in Z-lines in newborn hamster cardiomyocytes (Osinska and Lemanski, 1989). Vinculin is associated with DYSF (De Morrée et al., 2010), desmin (Osinska and Lemanski, 1989), and dystrophin (Kaprielian et al., 2000). Dystrophin and vinculin together provide mechanical support for sarcolemma (Tangney et al., 2013; Kaprielian et al., 2000).

Talin is a costameric protein (Figure 3) that predominantly interacts with the actin cytoskeleton. Talin isoforms 1 and 2 are required for the proper assembly of sarcomeres and myoblast fusion during the development of skeletal muscle (Senetar et al., 2007).

### 7.2 Sarcomeric proteins

Tubules are connected to the sarcomeric proteins (Furukawa et al., 2001; Liu et al., 2019). Sarcomeres are essential for the regulation of the ultrastructural maturation of neighboring organelles; however, it is unclear whether sarcomeres modulate the signal transduction pathways involved in cardiomyocyte maturation (Guo et al., 2021).

Protein α-actinin (ACTN2; Figure 3) is the main component of Z-lines and serves to cross-link F-actin (Guo et al., 2021). ACTN2-mutant cardiomyocytes showed less TATS content; the rest of the tubules were disorganized and showed dramatic dilatation of the tubular lumen. It was also revealed that ACTN2 regulates signal transduction and transcription beyond its canonical role as a structural protein (Guo et al., 2021).

Titin is a large, abundant protein that stabilizes the thick filaments and prevents the overstretching of sarcomeres (Granzier and Labeit, 2004). During contraction-relaxation cycles, titin maintains the structural organization of the sarcomere, as well as the organization of the SR and TATS (Granzier and Labeit, 2004). Developmental changes in the expression and location of titin were observed. In chicken skeletal muscle, the cross-striation of titin was observed at E14, concomitantly with an accumulation of SR cisternae near Z-line complexes where the first tubules appear (Flucher et al., 1993). A switch from higher to lower molecular mass titin isoform was observed during P5-P12 in developing hearts (Opitz et al., 2004). In mice suffering from dilated cardiomyopathy, the expression of higher molecular mass titin isoform resulted in a reduction of sarcomeric passive stiffness (Makarenko et al., 2004).

The initiating process of tubule growth could be associated with the maturation of Z-lines. Tcap (titin-cap, telethonin; Figure 3) is a small protein located on the Z-line periphery that defines titin borders and serves as a structural and anchoring center between the M-line and Z-lines (Valle et al., 1997). Tcap assembles later into Z-lines (Zhang et al., 2009). The level of Tcap expression is increased in cardiac and skeletal muscles during development (Mason et al., 1999). The interaction of Tcap with the tubular membrane is mediated via a mink subunit of K^+^ channels in cardiomyocytes (Furukawa et al., 2001). Tcap knockout in zebrafish skeletal muscle (Zhang et al., 2009) and in mouse cardiomyocytes (Ibrahim et al., 2012b) led to the development of the tubular system with a weaker or absent transversal organization. Ibrahim et al. (2012b) showed that Tcap interaction with the tubular membrane is particularly important in load-sensing. Under the condition of thoracic aortic constriction of Tcap knockout mice, the orifices of tubules in cardiomyocytes were damaged.

Obscurin is a Z-line protein that interacts with titin (Borisov et al., 2004). In the skeletal muscle of obscurin-deficient mice, the structure of longitudinal SR was damaged, and some alterations were observed with associated SR ankyrins (Lange et al., 2009). The docking, clustering, and lateral alignment of E-C coupling machinery, especially SR cisternae, in precise positions with newly formed myofibrils is mediated by obscurin. The delayed incorporation of obscurin into myofibrils coincided with the lateral fusion of newly formed myofibrils into large myofibrillar bundles (Borisov et al., 2004; Borisov et al., 2008). Obscurin mediates the interaction of longitudinal SR with the sarcomeric M-lines via myomesin (Lange et al., 2009).

Nexilin (NEXN; Figure 3) is an actin filament-binding protein that has been identified as a Z-line protein abundant in striated muscles. Global loss of NEXN leads to severe dilated cardiomyopathy, resulting in the death of all mice by P8 (Aherrahrou et al., 2016). The global cardiomyocyte-specific knockout of NEXN caused completely absent TATS in P10 even though the TATS was present at P5 in control mice. The building of periphery couplings between the sarcolemma and SR was impaired at E18.5 in NEXN cardiomyocyte-specific knockout of cardiomyocytes (Liu et al., 2019). However, it is not clear if the development of TATS was only delayed or completely abolished.

Myospryn (encoded by the cardiomyopathy-associated gene 5, CMYA5; Figure 3) is a recently described protein associated with the establishment of CRU architecture and positioning. The disorganization of TATS with the prevalence of longitudinal elements, along with the altered position of SR proteins (RYR2s, JP2s), was observed in CMYA5-knockout cardiomyocytes. Interestingly, administering a low level of CMYA5 in a minority of cardiomyocytes without impairing heart systolic function also caused transversal tubule disorganization. In P7 cardiomyocytes, RYR2 and CMYA5 were already colocalized in a striated pattern corresponding to Z-lines. If CMYA5 was ablated, a disrupted Z-line distribution of RYR2 was observed, which further demonstrates that CMYA5 is required for the positioning of RYR2 and jSR to Z-lines preceding the formation of tubules (Lu et al., 2022).

### 7.3 Intermediate filaments

More connections between TATS and myofibrils can be found among intermediate filaments.

The connection between the tubular system and Z-lines could be mediated by desmin (Lazarides, 1980). Intermediate filaments of desmin were described as 90 Å-wide, non-branched filaments, which form bundles running perpendicularly to the longitudinal axis of the myofibrils. These filaments seem to penetrate or encircle the Z-discs and form dense patches on sarcolemma and transversal tubules, penetrating the whole cytoplasm (Behrendt, 1977). An incomplete assembly of intermediate filaments could be seen in immature rabbit cardiomyocytes at P21, where the intermediate filaments connect only peripheral myofibrils, whereas, in the adult state, they cross the central mass and connect the opposite sides of the cell (Nassar et al., 1987). The arrangement of desmin in P3 hamster cardiomyocytes has been detected in the area of Z-lines and intercalated discs (Osinska and Lemanski, 1989). At P21, the desmin appeared to merge with TATS in rabbit hearts (Nassar et al., 1987). Desmin is concentrated in the periphery of Z-lines. It interlinks the myofibrils with sarcolemma (Capetanaki et al., 1997). Desmin keeps myofibrils in the register during cardiac hypertrophy (Watkins et al., 1987). Severe disruptions were observed in desmin-deficient mice, including loss of lateral alignment of myofibrils, damaged anchoring to the sarcolemma, and abnormal mitochondrial organization or nuclear position (Capetanaki et al., 1997). Desmin could be a strong candidate for the transversal alignment of TATS, as was proposed by Lazarides (1980). Thornell et al. (1997) described a detailed ultrastructure of desmin-deficient mice hearts in the early postnatal period (P5, P11) and 3–4 weeks; a dilated tubular system was observed. Colocalization of desmin and obscurin revealed (Borisov et al., 2004) initial lateral alignment of obscurin with myofibrils, whereas desmin only created diffuse filaments along the longitudinal axis.

Another intermediate filament, γ-actin (Figure 3), links sarcomeres with DGC in costameres (Ervasti, 2003), and γ-actin itself is connected to SR (Gokhin and Fowler, 2011). In a study of neonatal cardiomyocytes in culture, the function of DHPR channels was influenced by the assembly and disassembly of actin filaments. The addition of a stabilization agent led to a massive increase in Ca^2+^ current, whereas the addition of an agent for actin filaments disruption caused its decrease (Lader et al., 1999). The binding proteins α-actinin and dystrophin regulate the function of DHPR channels on the tubular membrane in cardiomyocytes (Sadeghi et al., 2002). The interaction of DHPRs on the tubular membrane with the actin cytoskeleton is regulated by the protein AHNAK in left ventricular cardiomyocytes (Hohaus et al., 2002).

### 7.4 Microtubules

Microtubules are non-sarcomeric filaments (Figure 3), oriented mostly along the longitudinal axis and running between myofibrils in cardiomyocytes (Watkins et al., 1987; Robison and Prosser, 2017). The microtubules interact with the TATS and SR (Zhang et al., 2014; Osseni et al., 2016). The microtubules grow from one Z-line to another, and they increase their density near the place where myofibrillogenesis and nuclei occur during hypertrophy (Watkins et al., 1987). The increase in microtubule density caused tubular remodeling in mice with induced cardiomyopathy (Zhang et al., 2014). Their relationship was revealed after the addition of colchicine, a microtubule depolymerizer. Its addition ameliorated the tubular remodeling. A similar response was observed in cultured cardiomyocytes. The distribution of JP2 was disorganized in failing hearts, although JP2 distribution was maintained if the colchicine was administered (Zhang et al., 2014). The microtubules of rat cardiomyocytes are modified and become stabilized during the postnatal period P2–P13 (Webster, 1997). Microtubules could play a mechanistic role in TATS development as stretch agents. A study by Meunier et al. (2009) on HeLa cells observed the interaction of microtubules and BIN1 via a linker protein. Overexpression of BIN1 caused the formation of tubules, which were closely aligned with microtubules. A driving force in tubulation induced by cholera toxins was mediated by molecular motors of microtubules (Day et al., 2015). These observations suggest that the interaction of microtubules with plasmalemmal patches enhances or allows the formation of tubules. Similarly, microtubules could have a role in tubulation mediated by DYSF protein; see Section 4 (Azakir et al., 2010).

## 8 Hypothesis: irregularity of tubular formation is a result of coupling to the developing myofibrils

**FIGURE 4:**
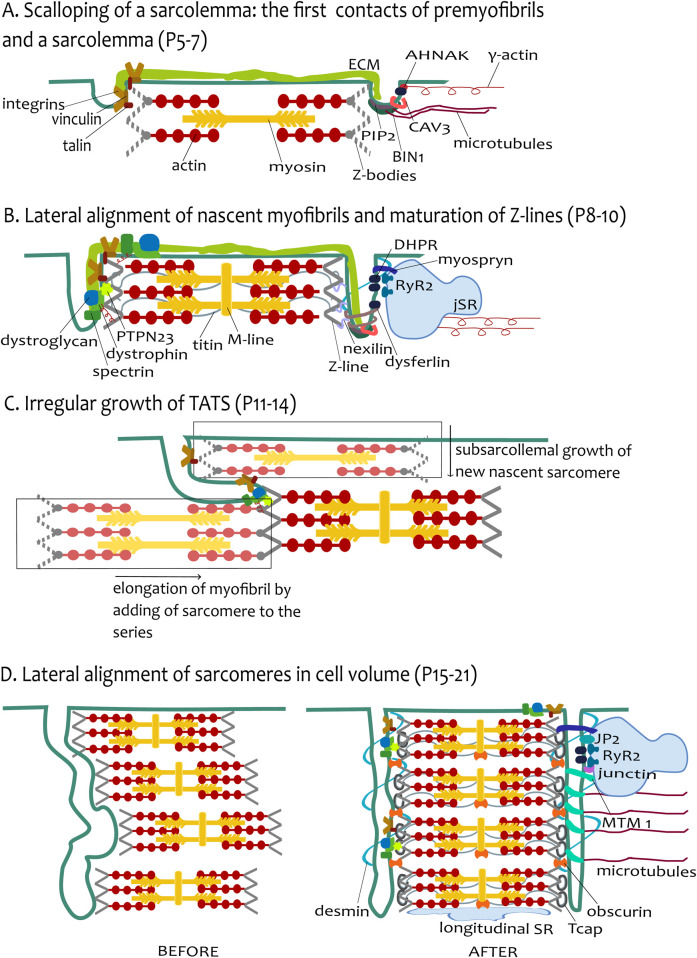
The hypothesis of tubular growth coupled with ongoing myofibrillogenesis. **(A)** Scalloping of sarcolemma: the first contact of premyofibrils and sarcolemma (P5–P7). The figure depicts the adhesion sites of the sarcolemma and the premyofibrils. For simplicity, one growing presarcomere of premyofibril is constructed from several Z-bodies connected with F-actin. The presarcomere is finished with myosin filaments among actin filaments. The environment of tubules is depicted differently in the left and the right tubules. The extracellular matrix (ECM) continues to both tubules. The left tubule emphasizes the talin–vinculin costamere units coupling with the Z-bodies. The right tubule shows the protein environment participating in the initial tubulogenesis as BIN1 assembled with PIP2 and CAV3 rings. The BIN1 can be pulled by microtubules. DHPR channels interacted with γ-actin filament through protein AHNAK. **(B)** Lateral alignment of nascent myofibrils and maturation of Z-lines (P8-P10). The tubules now grow like small transversal sarcolemmal invaginations, closely attached to the mature Z-lines. The sarcomere is now more mature (depicted by the created M-line), Z-bodies are transformed into Z-lines, and titin filaments are assembled, spanning across two Z-lines. The left tubule highlights the attachments with sarcomere Z-line proteins, strengthened by DGC costameric units (dystroglycan, spectrin, and dystrophin) pulled by γ-actin. Protein PTPN23 and nexilin improve the interaction of tubules with the Z-line. The right tubule shows the agents involved in tubulation (BIN1, dysferlin) with an emphasis on the CRU formation on tubules, provided by the assembly of cisternae of SR (jSR) with RyR2 channels facing tubular DHPRs. The tubule-jSR cleft is supported by the presence of myospryn. **(C)** Irregular growth of TATS (P11–P14). The image depicts the irregular growth of tubules by ongoing myofibrillogenesis. Transversal growth is induced by subsarcolemmal growth of new nascent sarcomeres (in lighter shades of original colors, in rectangles), whereas axial tubular growth is stimulated by the elongation of myofibril by the addition of sarcomere(s) in series. To simplify the image, only costameres are depicted as anchoring sites of tubules and sarcomeric Z-lines. **(D)** Lateral alignment of sarcomeres in cell volume (P15–P21). The left part (BEFORE) depicts the tubules and several sarcomeres before the final alignment, and the right part (AFTER) depicts the state of the final alignment of cell compartments. Desmin filaments provide the transversal scaffold for the coupled alignment of tubules and Z-lines, whereas microtubules support the axial alignment. The lateral alignment of myofibrils as the last stage of development is completed with Tcap assembly to the titin end and obscurin assembly among adjacent Z-lines and M-line-longitudinal SR interface. CRU formation is finished with the assembly of JP2 with junctin. The right tubule depicts the elongation of tubules induced by MTM1.

The present review and the most recent studies from iPSC and EHTs prove an extensive relationship between the development of contractile apparatus and TATS induced by mechanical and electrical stimuli. The question is how this process is mediated. Our hypothesis emphasizes TATS development coupled with ongoing myofibrillogenesis (Figures 1, 4).

The ongoing myofibrillogenesis during postnatal myocardial growth refers to the period when cardiomyocytes have an essential number of myofibrils for cardiac work, but the contractile apparatus needs to be enlarged to react to increased cardiac output. Hypertrophy of cardiomyocytes in terms of the growth of myofibrils is greatly influenced by the type of acting mechanical force. In the case of diastolic strain, cardiomyocytes add sarcomeres in series, and the cells are lengthened. In the case of cyclic strain from contraction, myocytes add myofibrils in a parallel way, and the cells are widening (Russell et al., 2010; Göktepe et al., 2010). Cardiac function is rapidly changed in the postnatal heart with increasing contraction force as well as increasing blood volume, which possibly leads to the dynamic change of diastolic strain and systolic wall stress.

In brief, the model of myofibrillogenesis proposes *de novo* myofibrillogenesis via the formation of premyofibrils to nascent myofibrils to mature myofibrils (Liu et al., 2012; Rhee et al., 1994). The assembly of premyofibrils is initiated either at the spreading ends or at the lateral sides of muscle cells. Premyofibrils are composed of small sarcomeres containing α-actinin-enriched Z-bodies attached to F-actin-enriched thin filaments. Z-bodies in adjacent fibrils align in nascent myofibrils, forming beaded Z-bodies that will metamorphose into mature Z-lines. Titin and thick filaments are first detected in the nascent myofibrils, whereas M-band proteins, like myomesin, are assembled later in mature myofibrils, presumably to help with the lateral alignment of thick filaments side by side. Tcap is another late-assembling protein, present only in mature Z-lines (White et al., 2014).

The leading mediators of TATS development are most likely the anchoring proteins between contractile apparatus and tubules, as we declared before; see section 7. When the functional demands rise with an increasing number of myofibrils shifting to the cell center, the first membrane invaginations coupled with premyofibrils are created. The assembly of mature contractile apparatus influences the development of tubules and the change of growth pattern from irregular into mostly transversal mode (Figure 4). The docking of sarcolemma to the Z-lines and to existing myofibrils, mediated by anchoring proteins, allows the growth of tubules into the cell center. The new myofibrils are formed in the proximity of peripheral sarcolemma, and the elongation of the tubules in the transversal direction is led by the anchorage to the already existing myofibril, which is moving closer to the cell center. Moreover, with the addition of new sarcomeres to the existing myofibril, the tubule growth also proceeds in the longitudinal direction. A combination of elongation of existing myofibrils with additions of new myofibrils might create a sparse tubular network with low-level organization, observed in a transitional phase of tubular network development.

The concomitant development of TATS, facilitated by ongoing myofibrillogenesis, can be described by three phases:

i) the first contact of premyofibril and sarcolemma;
ii) lateral alignment of nascent myofibrils, the maturation of Z-lines and sarcolemmal invaginations;
iii) lateral alignment of myofibrils in cell volume and final tubular arrangement.

### 8.1 The first contact of premyofibril and sarcolemma

Studies of rat and lamb heart development (Anversa et al., 1981; Brook et al., 1983; Hopkins et al., 1973) reported that myofibrils from earlier periods are sparse and show little organization, but later, with advancing development, there is an increasing number of myofibrils with well-defined striations. The myofibrils in rat ventricular cardiomyocytes at P5 were generally narrow, loosely distributed, and not well aligned with the longitudinal axis. The peripheral assembly of myofibrils was well documented in hamster cardiomyocytes at P5 (Messina and Lemanski, 1991). This state is in line with the expectation that there are some present myofibrils, but further postnatal myofibrillogenesis is ongoing. Moreover, the myofibrillogenesis is ongoing rather by elongation due to the addition of sarcomeres, while the number of myofibrils does not increase rapidly, as was observed in the early postnatal period (P0–P4) (Hirschy et al., 2006).

The initiation of adhesion site assembly for premyofibrils is facilitated by costameric proteins (especially integrins vinculin and talin). They also serve as nucleation sites for α-actinin accumulation, causing the assembly of premyofibril-associated Z-bodies (Sparrow and Schöck, 2009; Yang et al., 2015). Importantly, costameres are present in the tubular system as well (Kostin et al., 1998). An interesting phenomenon demonstrating the strength of structural attachment is the scalloping of membrane, which is induced by adhering sarcolemma with Z-lines, commonly featured in the myocardium of reptiles as well as mammals (Hirakow, 1970; Chiesi et al., 1981). The attachment of protocostameres with premyofibrils could also be a starting point for the anchoring of the tubular system and its first invaginations. The membrane curvature inducers such as BIN1 or CAV3 can aid in creating the orifice of the first tubules, showing like scalloping of the membrane (Figure 4A, P5–P7).

### 8.2 Lateral alignment of nascent myofibrils and maturation of Z-lines

Cardiomyocytes from the left ventricle of the mouse increased their width by lateral addition of myofibrils after P5 (Leu et al., 2001), which agrees with proceeding differentiation. Nascent myofibrils are displaced more to the interior, and the protocostameres mature into costameres in subsarcolemmal myofibrils and inter-Z-line bridges between internal myofibrils (Sparrow and Schöck, 2009). The dimensional restrictions due to the large nucleus centrally positioned in cardiomyocytes are slowly changing by binucleation during P4–P12 (Li et al., 1996). The space restrictions could partially explain the lateral addition of myofibrils and the increase in size, but only in sarcolemmal proximity (Figure 2A). Approximately from P9, the first tubules are visible as short transversal invaginations of the sarcolemma, which agrees with the ongoing maturation of Z-lines, such as titin positioning (Flucher et al., 1993). The Z-line protein nexilin also facilitates the connection between tubular invaginations (Liu et al., 2019). The tubular invaginations are more pronounced and reinforced by the presence of costameric proteins spectrin and dystrophin (Figure 4B, P8-P10) (Frank et al., 1994; Messina and Lemanski, 1991).

The transitional growth phase is marked by an outburst of developmental processes. The functional demands are growing: myofibrillogenesis is still ongoing. The M-line in sarcomeres appeared later, at P11, but only in 60% of the viewed sarcomeres (Anversa et al., 1981). During the transitional phase (at P10), the development of sarcomeres was not synchronous in all myofibrils of the cells, not even of adjacent sarcomeres of the same myofibril (Hopkins et al., 1973; Anversa et al., 1981). The combined development of mechanical force led to a dynamic model of myofibrillogenesis (Yang et al., 2016). The study demonstrated the dynamic addition of sarcomeres in live cells under stretch in a 3D culture of neonatal ventricular myocytes. Under longitudinal stretch, the elongation was observed in the longitudinal direction, but a lateral extension was also observed. The elongation consists of sarcomeres addition at the end and/or the insertion of sarcomeres in the middle of the existing myofibril. The formation of a new myofibril, using an existing one as a template, occurred during combined longitudinal and lateral stretches. The splitting of myofibrils was predicted by the model under lateral stretch (Yang et al., 2016). The acute rebuilding of sarcomeres and myofibrils in cardiomyocytes was observed after 3 h under static sustained stretch (Yang et al., 2016).

The question is whether the model is applicable also later in the postnatal period. The elongation of cardiomyocytes from the mouse's left ventricle was more pronounced after P12 (Leu et al., 2001). The attached tubules are probably pulled by developing myofibrils. The elongation of myofibrils and adding sarcomeres to the series of already-created myofibrils could explain the longitudinal growth of tubules (Figure 2B, P11–P14). The combination of ongoing maturation of subsarcomeric myofibrils with elongation of myofibrils supports the observation of the transitional phase of TATS development as an irregular network of tubules. Dysferlin and myotubularin are present to aid the elongation of tubules and the creation of networks. The formation of internal CRUs and indirect coupling of TATS with cisternae of SR around sarcomeres is further enabled by JP2 (Chen et al., 2013) and myospryn (Lu et al., 2022). Myospryn is localized to Z-lines and subsequently contributes to tethering cisternae of SR adjacent to these structures. TATS is subsequently formed close to cisternae, yielding organized, properly positioned dyads (Lu et al., 2022) (Figure 4B, P8-P10).

### 8.3 Lateral alignment of myofibrils in cell volume

One of the last assembled proteins during myofibrillogenesis is Tcap, which is inserted into the mature Z-lines (Zhang et al., 2009). Tcap in Z-lines facilitates the transversal organization of TATS (Zhang et al., 2009; Ibrahim et al., 2012b). Zhang et al. (2009) claimed that skeletal muscle movement facilitates the early invaginations of tubules by sensing the stretching force during twitch movement via Tcap. The expression of Tcap increased with increasing stretch force. One can speculate that the impulse for TATS development is then facilitated by increasing mechanical load and sensing it via lately assembled Tcap in the myofibrils, which are then pulled from the subsarcolemmal space deeper to the cell center.

Fully formed mature myofibrils were centrally positioned in the cell center, premyofibrils were closest to the cell periphery, and nascent myofibrils were located between the premyofibrils and the mature myofibrils in chicken embryonic cardiomyocytes (Rhee et al., 1994). The displacing of more mature myofibrils to the cell center also includes the lateral alignment of mature myofibrils at the Z-lines. The simultaneous lateral alignment of the sarcomeres was observed in skeletal muscle, with the rearrangement of tubules to the transversal orientation. Interestingly, locations with retained misalignment of the striation in the adult skeletal muscle were associated with longitudinal tubules (Franzini-Armstrong, 1991; Takekura and Kasuga, 1999). Thus, the regularity of the tubular system could be associated with the lateral alignment of myofibrils. That could also be true in cardiomyocytes, where the last phase of TATS development is marked by the alignment to pronounced transversal orientation (Figure 4C). Intermediate desmin filaments have the function of interlinking the adjacent myofibrils and keeping them in line together with the coupling of concurrent transverse tubules, as was previously proposed (Lazarides, 1980). The M-line protein obscurin is also involved in the lateral alignment of myofibrils in the bundles (Lange et al., 2009).

### 8.4 Possible contradictions of the hypothesis

Certainly, there are several contradictories of mutual connection—some authors declare sarcomerogenesis is ongoing near the polar ends of cells. Wilson et al. (2014) observed hypertrophy in mouse ventricular cardiomyocytes from P14 to maturity by electron microscopy. They proposed a model of sarcomere addition at the end of the myofibril, where it is connected to an intercalated disc. The tubule growth was given by the interaction of created sarcolemma folds, SR, and spectrin. They gave little hope for sarcomere addition elsewhere in cardiomyocytes. These results are contradictory to previously mentioned studies (Liu et al., 2012; Yang et al., 2016).

Perhaps the most recurring argument of our presented hypothesis is a synchronous observation of developing E-C coupling structures and myofibrillogenesis. Lainé et al. (2018) studied the development of E-C coupling machinery and myofibrillogenesis on iPSC-derived skeletal myocytes by electron and confocal microscopy. They observed the fully matured myofibrils after 22 days of differentiation, whereas the E-C coupling machinery was not placed. The question could be the maturation level of iPSC-derived skeletal muscle cells and their Ca^2+^ signaling because CRUs were atypically in the cell periphery and misaligned to their usual position in the A-I region. Moreover, there was no mechanical stimulation that could improve the overall iPSC maturity. However, in the more recent works from iPSC cells (Ronaldson-Bouchard et al., 2018; Godier-Furnémont et al., 2015), it appears that mechanical stimulation is a key factor to induce the growth of TATS. Moreover, the tubulation provided by curvature-inducing agents like BIN1 can be independent, but the coupling with other subcellular components, such as myofibrils, could explain why TATS is an organized, well-oriented network with determined density.

### 8.5 Research directions

The outlooks presented here might encourage mutual experimental approaches in the development of myofibrils and tubular systems. The development of the tubular system is not only the assembly of functional coupling between ion channels of the sarcolemma and sarcoplasmic reticulum but also a complicated process involving induction of membrane curvatures, membrane fusion and tubulation, protein trafficking, and docking ion channels probably orchestrated by the cytoskeleton and other proteins. The iPSCs crafted in mechanically stimulated engineered heart tissues (Godier-Furnémont et al., 2015) could be labeled with sarcolemma and TATS-sensitive dyes such as di-8-ANEPPS or FM-143FX together with labeled structures of myofibrils (such as RFP-tagged α-actinin, nexilin) by viral transfection and visualized by light sheet fluorescence microscopy in time-dependent manner to observe mutual development of myofibrils and TATS. A relationship between the magnitude of stimulation and TATS organization would be interesting to analyze.

Another approach could involve the stretching conditions of cardiomyocytes and dynamic sarcomere addition visualization, as was observed by Yang et al. (2016), using cardiomyocytes with present TATS such as iPSC-derived cardiomyocytes stimulated by thyroid hormone and dexamethasone (Parikh et al., 2017). These experiments might reveal whether the tubulation is independent of myofibrillogenesis or whether the assembly of structural units is coupled. Several models of myofibrillogenesis in embryonic state or *in vitro* were proposed*.* Whether the proposed models are also valid in the early postnatal period during the physiological hypertrophic response needs further analysis.

The possible synchronized and mutual development of structures to fulfill their physiological roles needs to be investigated thoroughly to understand the process

## 9 Conclusion

In this review, we summarized the postnatal development of the tubular system and contractile apparatus of cardiac cells. Cardiomyocytes are terminally differentiated cells pumping blood for the entire life of the organism. During the early period of life, cardiomyocytes need to not only work but also build the mechanics for future work. Each developmental process is usually observed separately. As one of the last specialized structures, the tubular system is developed in the *post-partum* period. While the tubular system is regularly spaced in adult cardiomyocytes, during postnatal growth, the tubular system is quite disorganized. A comparable phenotype was also observed during pathological conditions or in atrial cardiomyocytes. We summarize when and where these irregular tubular systems were observed. Because of the function of the tubular system in excitation and synchronization of Ca^2+^ waves in whole cell volume to all myofibrils, we hypothesized that the development of contractile apparatus—myofibrillogenesis—could influence the development of the tubular system. We summarize several interesting interactions, like PTPN23 or nexilin, which could be “the hot spots” in the development of TATS. Several authors also discussed this idea, but the overall picture of developmental processes has not yet been fully drawn.

## Abbreviations

ACTN2, α-actinin; BIN1, amphiphysin2; CAV3, caveolin 3; CICR, calcium-induced calcium release; CMYA5, myospryn, cardiomyopathy-associated gene 5; CRUs, Ca^2+^ release units; DGC, dystrophin-glycoprotein complex; DHPRs, dihydropyridine receptors; DYSF, dysferlin; E-C coupling, excitation–contraction coupling; EHT, engineered heart tissues; ECM, extracellular matrix; iPSCs, induced pluripotent stem cells; iPSC-CMs, cardiomyocytes derived from induced pluripotent stem cells; JP2, juncthophilin2; MTM1, myotubularin 1; NEXN, nexilin, P, postnatal; PIP2, phosphatidylinositol-4,5-bisphosphate; PTPN23, tyrosine phosphatase, nonreceptor type 23; RyRs, ryanodine receptors; SR, sarcoplasmic reticulum; TATS, transverse-axial tubular system; Tcap, titin-cap, telethonin.
